# The nuclear protein GmbZIP110 has transcription activation activity and plays important roles in the response to salinity stress in soybean

**DOI:** 10.1038/srep20366

**Published:** 2016-02-03

**Authors:** Zhaolong Xu, Zulfiqar Ali, Ling Xu, Xiaolan He, Yihong Huang, Jinxin Yi, Hongbo Shao, Hongxiang Ma, Dayong Zhang

**Affiliations:** 1 Provincial Key Laboratory of Agrobiology, Institute of Biotechnology, Jiangsu Academy of Agricultural Sciences, Nanjing 210014, China; 2 Department of Plant Breeding and Genetics, University of Agriculture, Faisalabad, Punjab, Pakistan

## Abstract

Plant basic-leucine zipper (bZIP) transcription factors play important roles in many biological processes and are involved in the regulation of salt stress tolerance. Previously, our lab generated digital gene expression profiling (DGEP) data to identify differentially expressed genes in a salt-tolerant genotype of Glycine soja (STGoGS) and a salt-sensitive genotype of Glycine max (SSGoGM). This DGEP data revealed that the expression (log2 ratio) of *GmbZIP110* was up-regulated 2.76-fold and 3.38-fold in SSGoGM and STGoGS, respectively. In the present study, the salt inducible gene *GmbZIP110* was cloned and characterized through phylogenetic analysis, subcellular localization and in silico transcript abundance analysis in different tissues. The functional role of this gene in salt tolerance was studied through transactivation analysis, DNA binding ability, expression in soybean composite seedlings and transgenic *Arabidopsis*, and the effect of GmbZIP110 on the expression of stress-related genes in transgenic *Arabidopsis* was investigated. We found that GmbZIP110 could bind to the ACGT motif, impact the expression of many stress-related genes and the accumulation of proline, Na^+^ and K^+^, and enhanced the salt tolerance of composite seedlings and transgenic *Arabidopsis*. Integrating all these results, we propose that GmbZIP110 plays a critical role in the response to salinity stress in soybean and has high potential usefulness in crop improvement.

Plants encounter various biotic and abiotic stressors during their life cycle. Growth and development is delayed when plants are exposed to extreme environmental conditions, such as high salt, drought, cold, and heat. Soil salinity affects large areas of cultivated land, which are naturally present in more than 100 countries worldwide^1^. The increasing and continued salinization of cultivated land threatens global crop production, especially in irrigated systems^2^ and has negative impacts on food security. The consequences are damaging in both socioeconomic and environmental terms. Increasing the salinity tolerance of crop plants may be an important contribution to the maintenance and stability of crop yields in salt-affected soils.

Plants can adapt to these adverse environmental conditions by regulating the expression of a large number of genes related to abiotic stress. A number of genes, including effector and regulatory genes, are reportedly involved in the alteration of their expression in response to stressors such as salinity^3^,^4^,^5^,^6^. Plants may adapt to these adverse environmental conditions by regulating of the expressions of a large number of abiotic stress-related genes. Stress-related genes are categorized into the following two major groups: genes that encode structural proteins and regulatory proteins, including transcription factors (TFs), and genes encoding signal-related protein kinases^7^,^8^. Stress-related TFs play important roles in the regulation of stress responses, such as in salt and drought tolerance. A single TF is sufficient to control the expression of multiple target genes involved in the responses to various severe stress conditions; this regulation occurs via binding to specific cis-acting elements in their promoters^9^.

Several families of TFs genes are reported in eukaryotes. The basic leucine zipper (bZIP) gene family is one of the most extensively investigated TF gene families; it is present in all eukaryotes. bZIP proteins contain a basic region consisting of ~16 amino acid residues that binds target DNA. This region contains a nuclear localization signal followed by an invariant N-X7-R/K motif, which contacts DNA. A second dimerization motif containing leucine repeats or other bulky hydrophobic amino acids creates an amphipathic helix known as a leucine zipper^10^. Other conserved domains, such as proline-rich, glutamine-rich and acidic domains, may function as transcriptional activation domains^11^,^12^. A set of 75 bZIPs in *Arabidopsis* was divided into ten groups according to sequence similarities in their basic regions^13^. Recently, bZIP proteins in angiosperms were divided into 13 groups based on the similarities in their bZIP domains and other conserved motifs^14^. Members of the bZIP family are regulators of important biological functions, such as organ and tissue differentiation, cell elongation, pathogen defense, the light response, osmotic control, energy metabolism, seed-related processes, and ABA-mediated stress responses^13^,^14^,^15^,^16^. bZIP proteins typically bind DNA as dimers. The formation of bZIP homo- and heterodimers provides huge combinatorial flexibility during the regulation of transcription^17^. In general, bZIP proteins prefer specific interactions^18^; such interactions are predicted to have important functions in gene regulation^19^. In *Arabidopsis*, bZIP protein members of group C and group S form specific heterodimers^13^,^17^. Surprisingly, almost no homodimerization was detected among members of group C and group S1; instead, rather specific and selective heterodimerization in observed among these bZIP proteins^19^.

Previously, digital gene expression profiling (DGEP) data were collected from cultivated and wild soybean in response to NaCl stress. Two bZIP genes were specifically expressed in wild soybean (*Glycine soja*); one was up-regulated (gnl|UG|Gma#S23068684 – GmbZIP110), and the other was down-regulated (gnl|UG|Gma#S5143834 – GmbZIP91). Four bZIP genes were expressed in both species. Of these, one was up-regulated (gnl|UG|Gma#S34818011 – bZIP110) and the other three were down-regulated (gnl|UG|Gma#S34818024 – bZIP60, gnl|UG|Gma#S34818016 – bZIP94, and gnl|UG|Gma#S34818015 – bZIP96)^20^. The up-regulated unigene was specific to wild soybean. The other unigene, expressed in both species, was GmbZIP110. GmbZIP110 is an ortholog of AtbZIP53 and belongs to group S^13^,^19^. AtbZIP53, along with AtbZIP1, is reported to reprogram primary C- and N-metabolism in salt-treated *Arabidopsis* thaliana roots; crosstalk between the two bZIP signaling pathways orchestrates salt-induced metabolic reprogramming in *Arabidopsis* roots, as reported elsewhere^21^. It is assumed here that GmbZIP110 forms a homodimer and confers salt tolerance in soybean.

In the present study, several stress-inducible bZIP TFs were cloned and analyzed in response to salt stress. One salt stress-inducible gene, *GmbZIP110*, was further characterized for its role in salt tolerance. The expression pattern of *GmbZIP110* in soybean seedlings under salt stress treatment and in specific tissues, its DNA binding ability, and the functional analysis of transgenic *Arabidopsis* plants overexpressing *GmbZIP110* were further investigated.

## Results

### Phylogenetic analysis of bZIP TFs and domain analysis and subcellular localization of GmbZIP110 protein

**Figure 1:**
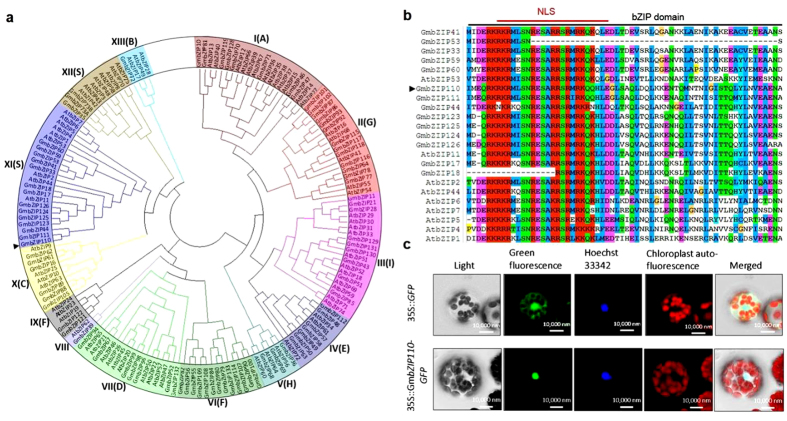
Phylogenetic analysis of the bZIPs and domain analysis and subcellular localization of the GmbZIP110 protein. (**a**) Phylogenetic tree containing full length soybean bZIPs, including GmbZIP110 (indicated by an arrow) and *Arabidopsis* bZIP proteins. A multiple sequence alignment containing the amino acid sequences of all bZIP proteins was performed using ClustalX2. The phylogenetic tree was constructed using the Neighbor-Joining method with the MEGA software. The bZIP transcription factors were divided into 13 subfamilies titled I–XIII. AtbZIP TFs were divided into 10 subfamilies titled A, B, C, D, E, F, G, H, I and S. GmbZIP110 belongs to the XI(S) subfamily, which contains 13 additional GmbZIPs and 9 AtbZIPs. (**b**) Domain structure of the GmbZIP110 subfamily XI(S): NLS, domain of putative nuclear localization signal under red bar, bZIP domain: basic region/leucine zipper motif of GmbZIP110 protein under black bar, the different background color of amino acid meaning different conserved sequences. (**c)** Subcellular localization of the GmbZIP110 protein in *Arabidopsis* protoplasts. A construct encoding a GmbZIP110-green fluorescent protein fusion (p35S::GmbZIP110-GFP) was created using the pJIT166-GFP vector. The GmbZIP110 was introduced without a termination codon, creating an in-frame fusion between the GmbZIP110 CDS and GFP. The fusion construct and the GFP control plasmid (p35S::GFP) were transformed into *Arabidopsis* protoplasts using PEG4000. The transformed *Arabidopsis* protoplasts were incubated for 18–24 h at room temperature and observed under a confocal fluorescence microscope. The nucleic acid stain Hoechst 33342 was detected using an excitation wavelength of 350 nm and an emission wavelength of 461 nm. Chloroplast auto-fluorescence was detected using an excitation wavelength of 480 nm and an emission wavelength of 685 nm. Scale bars = 10000 nm.

A comparative phylogeny of bZIP TFs gene families from soybean and *Arabidopsis*, including 99 full length GmbZIPs and 75 AtbZIPs, was divided into 13 subfamilies; these subfamilies were titled I–XIII (Fig. 1a). In a previous study, AtbZIP TFs were divided into 10 subfamilies; these subfamilies were titled A, B, C, D, E, F, G, H, I and S^13^. GmbZIP110 (Glyma08 g12170) belongs to the XI(S) subfamily. The consensus sequences of each group, based on an analysis of the AtbZIP proteins, are also shown.

The nucleotide sequence of *GmbZIP110* from *Glycine max* is identical to that from *Glycine soja*. The GmbZIP110 amino acid sequence contains one conserved bZIP domain that is 64 residues in length (Fig. 1b). The nuclear localization signal sequence RKRKRMLSNRESARRSRMRK (spanning amino acids 32–51) was found in the conserved bZIP domain of GmbZIP110. A GmbZIP110-green fluorescent protein (GFP) fusion construct and a control construct containing only GFP were created using the pJIT166-GFP vector; transcription was driven by the CaMV 35S promoter in both constructs. Constructs were introduced into *Arabidopsis* protoplasts by a PEG4000-mediated method. The cells were inspected with a confocal laser-scanning microscope to visualize the subcellular localization of the proteins. The nucleus was stained with Hoechst 33342. The GmbZIP110-GFP protein was detected only in the nucleus; the control protein was observed throughout the cell (Fig. 1c). These results indicated that GmbZIP110 was a nuclear protein.

### Differential transcript abundance analysis of *GmbZIPs* and *GmbZIP110*

**Figure 2:**
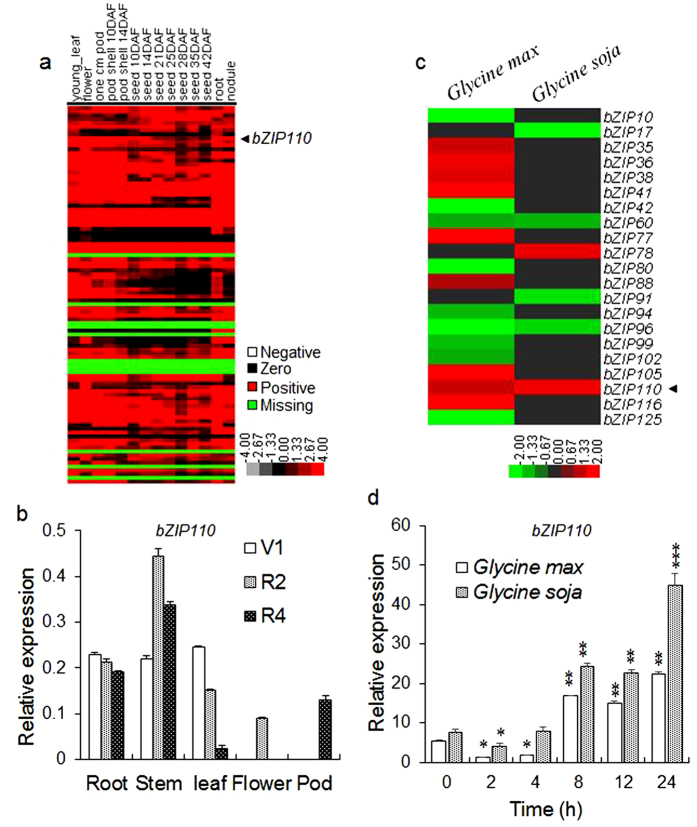
Transcript abundance profiles of GmbZIP genes. (**a**) In silico transcript abundance data for full length GmbZIP genes in young leaves, flowers, one cm pods, pod shells at 10 days after flowering (DAF), pod shells at 14 DAF, seeds at 10 DAF, seeds at 14 DAF, seeds at 21 DAF, seeds at 25 DAF, seeds at 28 DAF, seeds at 35 DAF, seeds at 42 DAF, roots and nodules was downloaded from SOYBASE. The legend (−4–4) indicates gene expression level. (**b**) Transcript abundance of GmbZIP110 in the roots, stems, leaves, flowers and pods of soybean at the first trifoliate (V1), full bloom (R2) and full pod (R4) stages. The experiments were carried out using qPCR. Different individual lowercase letters indicate significant differences as determined by one-way ANOVA followed by a Bonferroni post-hoc test. (**c**) Normalized transcript abundance (log_2_ ratio) of differentially expressed GmbZIP genes in a salt-tolerant genotype of *Glycine soja* (STGoGS) and a salt-sensitive genotype of *Glycine max* (SSGoGM) under 0 and 200 mM NaCl stress expressed as digital gene expression profiling (DGEP) data. The legend (−2–2) indicates the log_2_ ratio, which is the gene expression level under 200 mM NaCl treatment relative to that under 0 mM NaCl treatment. (**d**) Differential transcript abundance of GmbZIP110 in the roots of STGoGS and SSGoGM under 0 and 200 mM NaCl stress. P-values were calculated using Student’s t-test. *P < 0.05, **P < 0.01, and ***P < 0.001 compared with 0 h.

In silico transcript abundance of 99 *GmbZIPs* genes varied in the following samples: young leaves, flowers, one cm pods, pod shells at 10 days after flowering (DAF), pod shells at 14, seeds at 10 DAF, seeds at 14 DAF, seeds at 21 DAF, seeds at 25 DAF, seeds at 28 DAF, seeds at 35 DAF, seeds at 42 DAF, roots and nodules (Fig. 2a). The abundance of *GmbZIP110* transcripts was low in seeds and very high in nodule tissue.

Total RNA was isolated from roots, stems, leaves, flowers and pods of soybean at the first trifoliate (V1), full bloom (R2) and full pod stages (R4). RNA was reverse-transcribed to obtain cDNA for qPCR (real-time quantitative PCR) analysis. Transcript abundance was determined through qPCR analysis. The results showed that the relative abundance of *GmbZIP110* transcripts was highest in stem tissue at the R2 and R4 stages (Fig. 2b). The abundance of this transcript in the roots varied little at various growth stages and decreased with growth in the leaves. The *GmbZIP110* transcript was only expressed at the R2 and R4 stages in flowers and pods, respectively.

Digital gene expression profiling (DGEP) data were generated using a rigorous algorithm to identify differentially expressed genes (DEGs) in four different cDNA libraries^20^. The four libraries were constructed from control and NaCl-treated samples of a salt-tolerant genotype of *Glycine soja* (STGoGS) and a salt-sensitive genotype of *Glycine max* (SSGoGM). In response to salt stress, the expression of *bZIP* TFs was altered in STGoGS and SSGoGM (Fig. 2c). One gene, *GmbZIP78*, was up-regulated and two genes, *GmbZIP17* and *GmbZIP91*, were down-regulated specifically in STGoGS. One gene, *bZIP110*, was up-regulated in both accessions. The *GmbZIP110* gene was differentially up-regulated (log_2_ ratio) by 2.76-fold and 3.38-fold in SSGoGM and STGoGS, respectively.

To study the expression of *GmbZIP110* in response to salinity stress, the root samples were collected at 0, 4, 8, 12 and 24 h after NaCl treatment. The time-course qPCR results indicated that the transcript abundance of *GmbZIP110* decreased initially at 2 h, increased at 4 h and 8 h, was sustained at 12 h and finally increased at 24 h after NaCl application (Fig. 2d). The relative expression of *GmbZIP110* was increased by approximately 20-fold and 45-fold in the roots of *Glycine max* and *Glycine soja*, respectively, after 24 h of NaCl treatment.

### Transactivation analysis of GmbZIP110

**Figure 3:**
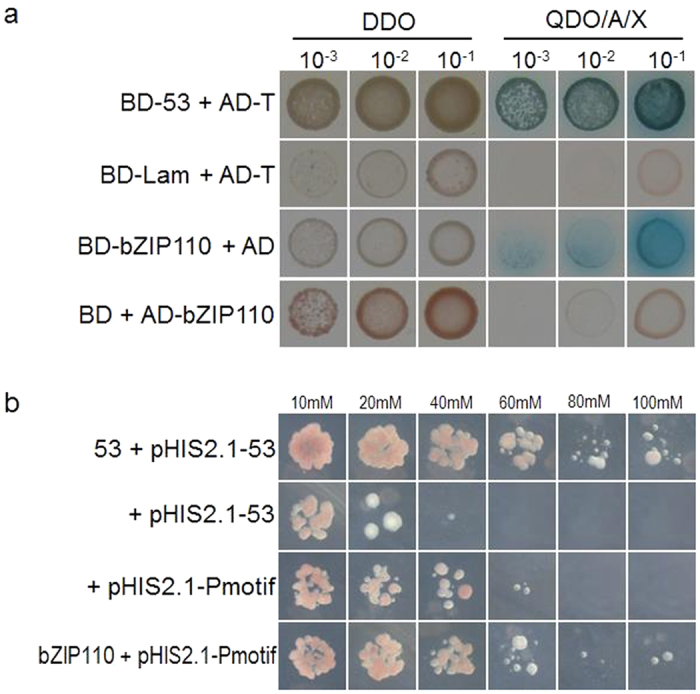
Transactivation and DNA-binding analyses of GmbZIP110. (**a**) Transactivation activity of GmbZIP110. Mated yeast growing on double dropout medium (DDO, SD/-Leu/-Trp) and quadruple dropout medium (QDO, SD/-Ade/-His/-Leu/-Trp) with 125 ng/ml Aureobasidin A and 40 μg/ml X-α-gal. Growth of cells harboring the BD-53/AD-T pair (positive interaction control), the BD-Lam/AD-T pair (negative control), BD + AD-GmbZIP110, and BD-GmbZIP110 + AD. Growth of the transformants on QDO/X/A; the blue color indicates that the corresponding gene has transactivation activity. (**b**) DNA-binding ability of GmbZIP110 was analyzed using a yeast one-hybrid system. The growth of cells harboring the effector and reporter plasmids pGADT7-Rec2-53 + p53HIS2.1 (positive control), pGADT7-Rec2 + p53HIS2.1 (negative control), pGADT7-Rec2 + pHIS2.1-P-motif (negative control) and pGADT7-Rec2-bZIP110 + pHIS2.1-P-motif. Growth of the transformants on triple dropout medium (TDO, SD/-Trp/-Leu/-His) with 3-AT indicates that the GmbZIP110 protein can bind to the corresponding cis-DNA element CACGTG.

Because the GmbZIP110 belongs to transcription factor of XI (S) subfamily, a yeast two-hybrid assay was carried out to analyze whether GmbZIP110 possessed transactivation function and to screen for interaction proteins Interestingly, all of the cross-combinations grew well on double dropout (DDO) synthetically defined medium (SD, SD/-Leu/-Trp). Cells containing the BD-GmbZIP110 vector and the AD empty vector could grow on quadruple dropout medium (QDO, SD/-Ade/-His/-Leu/-Trp) with Aureobasidin A and X-α-gal; colonies turned blue (Fig. 3a), which implied that GmbZIP110 had self-activation activity. These results indicate that GmbZIP110 has transcriptional activation function.

### Analysis of GmbZIP110 DNA binding ability using a yeast one-hybrid assay

bZIP proteins are known to bind to G-box elements^13^. Therefore, the DNA binding ability of the GmbZIP110 protein to a G-box element was examined using a yeast one-hybrid (Y1H) assay system (Clontech). Two tandem repeats of a G-box sequence (CACGTG) were synthesized and inserted into the pHIS2.1 vector, which contained the reporter gene *HIS3*. A minimal promoter was present downstream of the cis-elements and upstream of the *HIS3* gene. The reporter plasmid pHIS2.1, which harbored a double G-box motif plus the reporter gene *HIS3*, and the effector plasmid pGADT7-Rec2-GmbZIP110 were transformed together into the yeast strain Y187. The constructs pGADT7-Rec2–53 and p53HIS2.1 were used as a positive control. Negative controls where transformed with pGADT7-Rec2 and p53HIS2.1 or pGADT7-Rec2 and pHIS2.1-P-motif. Screening was carried out on triple dropout medium (TDO, SD/-His/-Leu/-Trp) containing 10, 20, 40, 60, 80 or 100 mM 3-amino-1, 2, 4-triazole (3-AT). The results showed that all of the transformants could grow on TDO medium containing a low concentration of 3-AT (Fig. 3b). Transformants harboring the effector plasmids pAD-GmbZIP110 and the reporter plasmid containing the G-box motif grew well on media with a high concentration of 3-AT medium. Growth on this medium was similar to that of the positive control, indicating that the GmbZIP110 protein could bind to the G-box motif.

### Salt tolerance response of soybean composite seedlings and transgenic *Arabidopsis* carrying the candidate gene *GmbZIP110*

**Figure 4:**
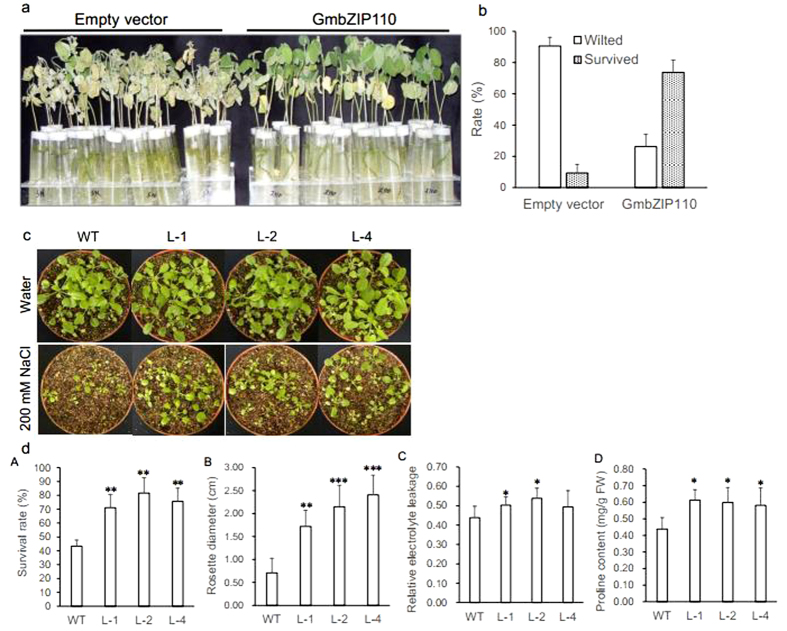
Effect of salt stress on soybean hairy roots and transgenic *Arabidopsis* plants overexpressing GmbZIP110. (**a**) Comparison of the salt tolerance response in hairy roots of soybean mosaic seedlings carrying an empty vector or a GmbZIP110 over-expression vector. Photographs were taken after one week of treatment with 200 mM NaCl. (**b**) Survival rate of the soybean mosaic plants in ‘**a**’ under saline conditions. (**c**) Response of the wild type and three transgenic *Arabidopsis* lines to saline and non-saline conditions. (**d**) d-A, Survival rate, d-B, rosette diameter, d-C, relative electrolyte leakage and d-D, proline content of the transgenic *Arabidopsis* plants in ‘**c**’ under saline conditions. P-values were calculated using Student’s t-test. *P < 0.05, **P < 0.01, and ***P < 0.001 compared with the empty vector or WT.

As the expression of *GmbZIP110* can be induced by NaCl stress application, so the function of GmbZIP110 was investigated through its construction in a plant over-expression vector and generation of soybean composite seedlings via *Agrobacterium rhizogenes* mediated transformation. Performance of the soybean mosaic seedlings was examined under NaCl treatment. Figure 4a showed that the salt tolerance response of composite seedlings carrying the candidate gene *GmbZIP110* was improved compared to control seedlings under 200 mM NaCl stress. Most of the transgenic *GmbZIP110* composite seedlings (73.7%) survived, with some wilted leaves; 91% of the transgenic composite seedlings carrying the empty vector died (Fig. 4b).

The function of GmbZIP110 in plant under salt stress was also investigated through generation of transgenic *Arabidopsis* plants. Under normal condition, all the transgenic lines showed no significant phenotypic and survival rate differences with the wild type (WT) plants (Fig. 4c). Four-week-old transgenic *Arabidopsis* seedlings were treated with 200 mM NaCl for one week. WT seedlings were used as control. Seedlings from transgenic lines L-1, L-2 and L-4 showed improved growth compared to the WT (Fig. 4c). The survival rate, rosette diameter, relative electrolyte leakage and proline content of transgenic lines L-1, L-2 and L-4 were significantly higher than those of the WT, one exception, L-4, showed a non-significant increase in relative electrolyte leakage (Fig. 4dA–4dD). Proline synthesis and accumulation is involved in the responses to drought, salt stress, and osmotic stress^22^,^23^,^24^. These results demonstrated that the overexpression of *GmbZIP110* could improve the response to salinity stress in plant.

### Na^+^ and K^+^ content in transgenic *Arabidopsis* seedlings over-expressing GmbZIP110 gene under 150 mM NaCl stress

**Figure 5:**
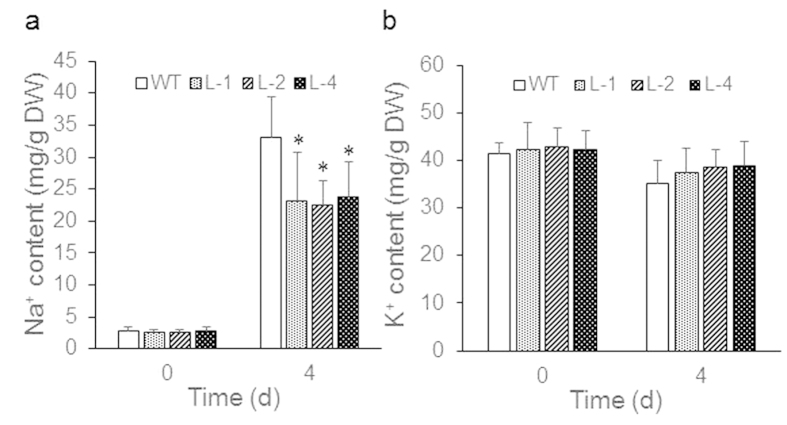
Na^+^ and K^+^ ion uptake by two-week-old wild type and transgenic *Arabidopsis* plants overexpressing GmbZIP110 after 0 and 4 days of NaCl treatment. (**a**) Na^+^ ion uptake assay. (**b)** K^+^ ion uptake assay. P-values were calculated using Student’s t-test. *P < 0.05 compared with 0 d.

Since *GmbZIP110* could improve salinity tolerance response in composite seedlings and transgenic *Arabidopsis* plants, it may be through regulating the Na^+^ absorption. Two-week-old transgenic *Arabidopsis* seedlings grown on ½ MS culture medium were transferred to ½ MS medium containing 150 mM NaCl. WT seedlings were grown as a control. After four days of treatment, Na^+^ and K^+^ contents were determined. The dry matter of WT seedlings and the three transgenic lines, i.e., L-1, L-2 and L-4, contained ~3 mg Na^+^ per g of dry weight at 0 d after the application of NaCl treatment (Fig. 5a). WT plants took up 33 mg Na^+^ per g of dry weight. This uptake was significantly greater than that of the transgenic lines, which took up ~23 mg Na^+^ per g of dry weight at 4 d after the application of NaCl treatment. The uptake of K^+^ was similar in the WT and the transgenic lines at 0 and 4 d after NaCl treatment; WT seedlings and the transgenic lines took up ~42 and 37 mg K^+^ per g of dry weight, respectively (Fig. 5b). These results indicate that GmbZIP110 has the fuction of regulating absorption of Na^+^.

### Expression analysis of 13 stress-related genes in transgenic *Arabidopsis* seedlings over-expressing *GmbZIP110*

**Figure 6:**
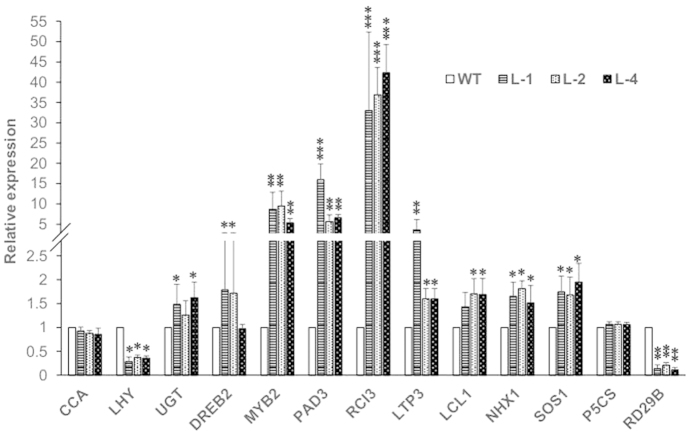
Relative transcript abundance of 13 stress-related genes in the leaves of two-week-old transgenic *Arabidopsis* seedlings over-expressing the *GmbZIP110* gene. The *Actin* (NM_112764.3) gene was used as an internal control. P-values were calculated using Student’s t-test. *P < 0.05, **P < 0.01, and ***P < 0.001 compared with WT.

The GmbZIP110 might have improved plant stress tolerance through regulation of downstream genes. We selected 13 stress responsive genes for further analysis. The relative expression levels of stress-related genes were determined by qPCR. cDNA from the leaves of two-week-old plants grown on ½ MS medium was used as templates. The transcript abundance of nine stress-related genes, *UGT*, *DREB2*, *MYB2*, *PAD3*, *RCI3*, *LTP3*, *LCL1*, *NHX1* and *SOS1*, increased significantly in the three transgenic *Arabidopsis* lines compared with that in WT; the transcript abundance of two genes, *LHY* and *RD29B*, decreased significantly (Fig. 6). The relative expression of *RCI3* in the three transgenic *Arabidopsis* lines was ~40. The relative expression of *CCA* and *P5CS* was similar in transgenic and WT plants. *RCI3* encodes a peroxidase and its overexpression conferred dehydration and salt tolerance^25^. *UGT*, *PAD3* and *LTP3* were involved in ABA responses^26^,^27^,^28^. *DREB2* expression does not activate downstream genes under normal growth condition, however, its overexpression leads to drought stress tolerance and slight freezing tolerance^29^. *MYB2* and *LCL1* have been found to be responsive to stress and/or ABA^30^. NHX1 and SOS1 are Na^+^/H^+^ exchanger in *Arabidopsis* and previous investigations have suggested them to play an important role in salt tolerance^31^,^32^. *LHY* and *RD29B* have been found to be responsive to stress and/or ABA^33^. These results indicated that GmbZIP110 regulated a common set of genes as well as specific sets of genes for NaCl stress tolerance.

## Discussion

Plants have the capability to respond and adapt to adverse environmental stressors, such as drought, cold, and salt, through physiological, biochemical, molecular and cellular processes^34^,^35^. These environmental stressors alter the expression of a large number of genes in plants. The expression of *bZIP* gene family members was also altered; these proteins regulate important biological functions, including biotic and abiotic stress responses^13^,^14^,^15^,^16^. These *GmbZIP* TFs showed differential expression in wild and cultivated soybean under normal and NaCl stress (Fig. 2c). GmbZIP110 is one of 99 full-length soybean bZIPs (Fig. 1a) that is strongly induced by salt (Fig. 2b–d), and its transcript levels are highest in nodule tissue and second highest in roots (Fig. 2a). Previous studies identified 47 full-length *GmbZIP* genes, which were divided into ten groups based on cluster analysis and a comparison with *Arabidopsis bZIP* genes^36^, Here, we identified 99 full-length *GmbZIP* genes that are divided into 13 groups; these findings are identical to those of Corrêa *et al.* (2008). Subgroups F and S are each divided into two groups; subgroup F and subgroup S contain groups VI and IX and groups XI and XII, respectively. A new subgroup, VIII, contains only one AtbZIP protein. This protein, AtbZIP62, does not belong to a group in Jakoby’s study. Subgroup VIII also contains one GmbZIP protein, GmbZIP39. This change is dependent on genetic distance.

*Arabidopsis* plants overexpressing *GmbZIP110* showed a slightly greater salt tolerance compared to WT plants (Fig. 4d). This finding was consistent with the expression of *OsbZIP23*, which was induced by dehydration, high salt stress and exogenous ABA application^37^, Improved plant stress tolerance was reported in plants over-expressing *OsbZIP23*. A recent study showed that the over-expression of SCOF-1 (soybean cold-inducible factor-1), which is a cold-inducible zinc finger protein gene from soybean, induced the expression of cold-regulated genes and enhanced cold-tolerance in transgenic plants. However, SCOF-1 did not bind directly to either CRT/DRE or ABRE. SCOF-1 up-regulated ABRE-dependent gene expression and mediated cold-regulated gene expression via protein-protein interaction with SGBF-1 (soybean G-box binding factor 1), a G-box binding bZIP transcription factor from soybean^38^. Similarly, expression of the intact AREB1 gene on its own is insufficient for the expression of downstream genes under normal growth conditions. No alterations in growth phenotype were observed between the wild type and AREB1 over-expressing plants. Plants over-expressing AREB1QT, an activated form of AREB1, showed ABA hypersensitivity and enhanced drought tolerance^39^. The over-expression of GmbZIP110 was sufficient for the regulation of downstream genes (Fig. 6). GmbZIP110 activated reporter genes when used as bait with prey or empty AD plasmids, indicating the presence of a transcriptional activation domain in in this protein (Fig. 3a).

The transgenic plants over-expressing GmbZIP110 showed higher survival rate, increased rosette diameter, relative electrolyte leakage and proline content compared with those of WT plants (Fig. 4d). These results especially higher relative electrolyte leakage together with the fact that transgenic *Arabidopsis* plants have taken-up significantly less Na^+^ ion while no significant change in K^+^ ion uptake (Fig. 5), might resulted in enhancement of salt tolerance of transgenic *Arabidopsis* lines (Fig. 4c). This suggests that GmbZIP110 maybe participates in other ion mediation, such as Ca^2+^, Amtmann^40^ reported increasing the concentration of Ca^2+^ in intercellular leads to less absorption of Na^+^. So GmbZIP110 might mediate some other harmless ion up-taking, thus to reduce the toxicity of high concentration of NaCl.

The differential expression of stress-related genes in transgenic *Arabidopsis* plants in comparison with WT was evident in the present study (Fig. 6). These genes included *UGT*, *DREB2*, *MYB2*, *PAD3*, *RCI3*, *LTP3*, *LCL1*, *NHX1* and *SOS1*, which significantly increased in relative transcript abundance. *LHY* and *RD29B* significantly decreased in relative transcript abundance. DREB2A acts as a trans-acting factor in the signal transduction pathway under dehydration conditions; expression of this protein was induced by dehydration^41^. *RD29B* is a well-studied dehydration-responsive gene; it contains at least one cis-acting element that is involved in the ABA response. The expression of *RD29B* was markedly increased under dehydration, high salt, and low temperature conditions^42^. In the present study, the expression of *RD29B* was significantly reduced. The gene *P5CS* (delta 1-pyrroline-5-carboxylate synthase), which catalyzes the rate-limiting step in the proline biosynthesis pathway, is induced by salt stress, drought, and ABA^43^. The expression levels of *P5CS* were similar in transgenic *Arabidopsis* and the WT. *RCI3* encodes a peroxidase and its overexpression conferred dehydration and salt tolerance^25^. *UGT*, *PAD3* and *LTP3* were involved in ABA responses^26^,^27^,^28^. *DREB2* expression does not activate downstream genes under normal growth condition, however, its overexpression leads to drought stress tolerance and slight freezing tolerance^29^. *MYB2* and *LCL1* have been found to be responsive to stress and/or ABA^30^. NHX1 and SOS1 are Na^+^/H^+^ exchanger in *Arabidopsis* and previous investigations have suggested them to play an important role in salt tolerance^31^,^32^. *LHY* and *RD29B* have been found to be responsive to stress and/or ABA^33^. So we indicated that GmbZIP110 might be involved in ABA dependent pathway and also related to ion transport.

In transgenic plants overexpressing GmbZIP110, the expression of these stress-responsive genes was up-regulated (Fig. 6). Salt stress induced the expression of *GmbZIP110*, and its over-expression further up-regulated several genes involved in salt tolerance in transgenic *Arabidopsis* plants. These findings demonstrate that GmbZIP110 plays a role in salt tolerance in soybean. No differences were found between the nucleotide sequences of this gene in *Glycine mas* and *Glycine soja*, (Fig. 1b); however, the expression levels of this gene are obviously different (Fig. 2c). This discrepancy may arise from differences in the promoter regions. The promoter is a critical area containing cis elements that regulates gene expression levels^44^,^45^. However, a closer look at the expression of *GmbZIP110* and stress-responsive genes at different developmental stages reveals more about the relationship between the function of GmbZIP110 and the performance of transgenic plants.

## Materials and Methods

### Plants and growth conditions

The present study followed published procedures for the generation of plant materials, growth conditions, total RNA isolation, reverse transcription, Tag library construction and sequencing, data analysis, qPCR and overexpression analyses^20^.

STGoGS and SSGoGM seedlings were grown in a greenhouse in pots containing nutritional soil, vermiculite and pearlite at a 1:1:1 ratio. Three-week-old soybean seedlings were up-rooted, and the roots were washed with distilled water. Seedlings of each accession were divided into two groups and placed in separate glass beakers filled with water. Roots from one group were placed in a 200 mM NaCl solution; roots from the second group were place in water as a control. Root samples were collected after 0, 4, 8, 12 and 24 h of NaCl treatment. Total RNA was isolated and reverse transcribed to obtain cDNA, which were used as templates for PCR.

### Identification of salt-responsive GmbZIP genes

Digital gene expression profiling (DGEP) data were generated using a rigorous algorithm developed to identify differentially expressed genes (DEGs) among control and NaCl-treated samples^46^. Genes with a false discovery rate (FDR)^47^ less than 0.05 and a fold change log2 ratio (stress/normal) either greater than +1 or less than −1 were considered to be differentially expressed genes. A new bZIP TF with significant differential expression across treatments and accessions was identified from the DGEP data.

### Phylogenetic and in silico analyses

The peptide sequences of GmbZIPs and AtbZIPs were mined bioinformatically from http://www.igece.org/Soybean_TF/ and http://www.ncbi.nlm.nih.gov/. Sequences were aligned using ClustalX 1.8 [ftp://ftp.ebi.ac.uk/pub/software/clustalw2]. The untreated phylogenetic tree was constructed using the neighbor-joining method with MEGA version 5^48^.

The expression data (Supplementary Table S1) for GmbZIP TFs in different tissues was downloaded from “soybase (http://soybase.org),” analyzed in silico and displayed using Java-Treeview software.

### Sequence retrieval, primer design, amplification, sequencing and domain analyses of GmbZIP110

Nucleotide and amino acid sequences for Glyma08 g12170 (GmbZIP110) were downloaded from phytozome v10. Forward and reverse primers, (5′-ATGGCTTCTCCTGGTGGAAGT-3′ and 5′-TCAATACATCATCAACATGTCATTG-3′), respectively, were designed and synthesized by Invitrogen^TM^. GmbZIP110 was PCR amplified using cDNA from *Glycine max* and *Glycine soja* as templates. The amplicons from both species were cloned and sequenced by GenScript.

Domain analysis and NUCDISC discrimination of nuclear localization signals (NLS) were performed for the peptide sequences using online tools at http://wolfpsort.org/.

### *Arabidopsis* protoplast isolation and sub-cellular localization

*Arabidopsis* protoplasts were isolated as previously described^49^. The CDS of GmbZIP110 was inserted into the pJIT166-GFP vector without a termination codon to create an in-frame fusion between GmbZIP110 and GFP. The fusion construct (p35S::GmbZIP110-GFP) and a control construct (p35S::GFP) were introduced into *Arabidopsis* protoplasts by PEG4000 method as Abel *et al.* described^50^. After incubation of transformed *Arabidopsis* protoplasts in culture solution for 18–24 h at room temperature, Hoechst 33342 was added to 10 μM final concentration and incubated at room temperature for more than 3 hours. Confocal imaging was performed using a confocal microscopy (Zeiss, LSM510 Meta, Carl Zeiss AG) and a ×40 lens. Fluorescence of GFP was measured at 495–545 nm. Hoechst 33342 was measured at 350–461 nm. Chloroplast auto-fluorescence was measured at 480–685 nm.

### Real-time Quantitative PCR

RNA from STGoGS and SSGoGM roots under 200 mM NaCl stress treatment and control conditions were used for qPCR analysis. Total RNAs were isolated using TRIzol^®^ reagent (Invitrogen & Co.), following the manufacturer’s instructions. Sample preparation and qPCR analysis was performed using SYBR^®^ Premix Ex Taq^TM^ (Perfect Real Time) in a Roche Light Cycler 2.0 with Light Cycler software (build 4.1.1.21), (LightCycler^®^ Carousel-based System, F. Hoffmann-La Roche Ltd, Germany). A 10 μl reaction consisted of 5 μl of SYBR^®^ Premix Ex Taq^TM^ (TaKaRa Bio Inc., Shiga, Japan, http://www.takara-bio.Com/), 0.8 μl of each primer (forward and reverse, at 10 mM), 1 μl of reaction product (cDNA) from RT-PCR as template and 2.4 μl of dH_2_O. The soybean gene *Gmactin* (accession number: NM_001250673, forward primer, 5′-CGGTGGTTCTATCTTGGCATC-3′; reverse primer, 5′-GTCTTTCGCTTCAATAACCCTA-3′) was used as an internal control for the detection of the expression of the target GmbZIP110. An equal amount of cDNA template was used for each sample, including the internal control. qPCR amplification conditions were as follows: an initial denaturation step for 30 s at 95 °C, and 35 quantification cycles consisting of denaturation for 5 s at 94 °C, annealing for 10 s at 58 °C, and extension for 30 s at 72 °C. A melting curve analysis was performed to confirm the specificity of the PCR products. Similar results were obtained from the relative gene expression data using the change in threshold cycle (DCt) method as described by Winer. Specific gene expression levels were considered not applicable (N/A) if Ct (gene) values were >30 or <15. The qPCR analysis was repeated in three independent experiments.

### Transactivation and dimerization analyses of GmbZIP110 through yeast two-hybrid assays

The Matchmaker^TM^ Gold Yeast Two-Hybrid System (Clontech) was used for the yeast two-hybrid experiments; assays were performed following instructions given in the Matchmaker^TM^ user manual and the Yeastmaker^TM^ Yeast Transformation System 2 user manual. The GmbZIP110 DNA sequence was cloned into the pGBKT7-53 DNA-BD vector (which contains the GAL4 DNA binding domain, BD) as bait and into the pGADT7-T AD vector (which contains the GAL4 DNA activity domain, AD) as prey. The bait vector BD-GmbZIP110 was transformed into the yeast strain Y2HGold, and the prey vector AD-GmbZIP110 was transformed into the yeast strain Y187. The two yeast strains were combined in a flask and shaken slowly at 30–50 rpm at 30 °C for 20–24 h. The two strains mated, resulting in diploid cells. The mated yeasts were incubated on DDO and QDO/X/A plates at 30 °C for 2 days.

### DNA binding analysis of GmbZIP110 through a yeast one-hybrid assay

Two tandem repeats of a G-box cis-element sequence (CACGTG) were synthesized (Invitrogen, Shanghai, China) and annealed. The annealed double-stranded DNA included E*coRI* and X*baI* restriction sites at each end. The annealed double-stranded DNA was double-digested with the restriction enzymes E*coRI* and X*baI*. The fragment was cloned into the pHIS2.1 reporter plasmid, which contained the nutritional reporter gene *HIS3*. A minimal promoter was present downstream of the cis-elements and upstream of the *HIS3* gene. The sequence of the insert was confirmed by sequencing. An effector plasmid was constructed independently. The coding region of GmbZIP110 was cloned into the DNA activation domain vector pGADT7-Rec2. Transformation and screening analyses were carried out according to the manufacturer’s instructions (Clontech). Both the effector and reporter plasmids were transformed into Y187 yeast cells. The transformants were selected on SD/-Trp-Leu media. The transformed yeast cells were plated onto SD/-Trp-Leu-His media containing 3-AT, and cell growth was examined.

### Development of mosaic soybean seedlings

An *Agrobactarium rhizogenes*-mediated soybean genetic transformation system was used to introduce the expression cassette into the soybean variety ‘Dongnong 690′. Soybean seeds were sown in pots containing vermiculite. Upon the emergence of cotyledons, healthy and robust seedlings were selected for transformation.

Agrobacteria carrying the *Gm*bZIP110 gene were streaked onto a LB plate containing kanamycin and pre-cultured at 28** **°C for two days. A single colony was used to inoculate liquid LB medium containing kanamycin as selectable marker. The culture was grown overnight at 28 °C with shaking at 180 rpm. The culture was centrifuged at 5,000 rpm for two min at room temperature to harvest the bacteria. The pellet was suspended gently in 10 mM MgCl_2_ solution and washed twice. The OD_600_ of the final bacterial suspension was adjusted to 0.4–0.6.

Young seedlings with unfolded cotyledons were infected at the cotyledonary node and/or the hypocotyl with *Agrobacterium rhizogenes* carrying the pCXSN-GmbZIP110 construct. The injection point was covered immediately after inoculation with a wet vermiculite/soil mixture. The infection sites were kept in a high humidity environment. Water was applied as required. The seedlings were grown in a greenhouse at 25 °C with a 12 h photoperiod and approximately 60% humidity. Hairy roots were induced at the inject point after one week; the roots had developed to 2–3 cm in length at one week after inoculation. In this way, the hairy roots developed at 3 weeks after sowing. The main roots were removed when the emerged hairy roots could support the plants^51^. Composite seedlings with one hairy root were transferred to 100 ml glass tubes containing ½ strength Hoagland medium^52^. The glass tubes containing composite soybean seedlings were placed in a growth chamber set at 25/22 °C day/night temperature with a 12 h photoperiod for approximately one week. The Hoagland solution in the glass tubes was replaced every other day. Once the hairy roots had grown to the bottom of the tube, the transformation of hairy roots was verified through PCR analysis. PCR was performed using a forward primer that aligned to the CaMV35S promoter (5′-CAATCCCACTATCCTTCGCAAGACC- 3′), a GmbZIP110 gene-specific reverse primer and genomic DNA from the hairy roots as a template. One set of soybean composite seedlings carrying the pCXSN empty vector was prepared for use as a control. Transgenic hairy roots of composite seedlings were immersed in ½ strength Hoagland solution containing 200 mM NaCl and grown for one additional week in a growth chamber. The Hoagland solution containing salt was replaced every other day. The data were recorded after one week of stress treatment. The experiment was repeated 3 times.

### Development of transgenic *Arabidopsis*

The GmbZIP110 CDS was cloned into the plant expression vector pCXSN using TA cloning. The sequence of the construct was confirmed by PCR and sequencing. The gene was driven by a double CaMV35S promoter. The resulting plasmids were transformed into the *Agrobacterium tumefaciens* strain EHA105, which was used to transform *Arabidopsis* Columbia-0 plants via the flower dip method. Transformed EHA105 was cultured overnight at 28 °C with shaking at 150 rpm; LB medium containing rifampicin (50 mg/L) and kanamycin (50 mg/L) was used. When the OD_600_ of the EHA105 culture reached 1.0, the culture was centrifuged at 5000 rpm for 2 min. The pellet was suspended and washed twice with the same volume of 5% sucrose. Finally, 0.1% SILWET L-77 was added. A drop of solution was placed on mature flower buds with unopened petals. The plants were kept in a high humidity environment in the dark for one day. Mature pods of treated Arabidopsis were harvested, and seeds were sown on selected medium containing 100 mg/L hygromycin B. Five transformed resistant plants were transplanted into green house, and confirmed by PCR. Line 1, 2, 4 (L-1, L-2, L-4) were GmbZIP110 overexpressing plants, these three lines were continued to develop and homozygous transgenic *Arabidopsis* lines were used for further analysis.

Seeds of WT and transgenic *Arabidopsis* were planted in 8 cm pots containing vermiculite and nutritional soil (3:1). Pots were placed in growth chamber set at 22/20 °C day/night temperature with a 12 h photoperiod. Water was applied as required. Four-week-old seedlings were supplied with 10 ml of 200 mM NaCl solution every day for one week. For one set of seedlings, 10 ml of water was applied; these plants were used as a control. After one week of treatment, rosette diameter, survival rate, relative electrolyte leakage and proline content were measured. The experiments were repeated independently at least three times.

### Relative electrolyte leakage

Relative electrolyte leakage was determined following methods described previously^53^. The leaf segments from three to six seedlings (0.1 g) were vacuum infiltrated in deionized water for 10 min and maintained in water for 2 h. The conductivity (C1) of the obtained solution was measured using a DDS-307A conductivity detector (Leici, China). The solution was boiled for 15 min and cooled to room temperature; the conductivity (C2) of the resulting solution was also measured. The relative electrolyte leakage was computed as fraction of C1–C2 (C1/C2). Each data point represents an average of three independent experiments. The data were subjected to statistical analysis using the t-test.

### Free proline content measurements

The free proline content of plants was determined following methods described previously^54^. Plant tissues (0.1 g) were homogenized in 1 ml of 3% sulfosalicylic acid using a pre-washed mortar and pestle. The mixture was centrifuged at 12000 rpm for 15 min at 4 °C. A 0.2 ml of the extract was placed in a test tube, and 0.2 ml each of glacial acetic acid and ninhydrin were added. The reaction mixture was boiled at 100 °C in a water bath for 1 h. After reaction mixture was cooled, 0.4 ml of toluene was added. After thorough mixing, 0.3 ml of chromospheres containing toluene was separated for the measurement of absorbance at 520 nm. Measurements were performed against toluene blank using a spectrophotometer (SHIMADZU, UV-2550). Each data point represents the average of three plant samples. Two independent experiments were performed.

### Determination of Na^+^ and K^+^ contents

Seeds of transgenic and WT *Arabidopsis* were sterilized with 75% ethanol for 30 s, followed by an 8 min incubation with 0.5% sodium hydrosulfite with 0.05% Tween 20. The seeds were rinsed three times with sterile distilled water. Fifty seeds were sown on ½ MS medium containing 0.8% agar in coated 9 cm petri dishes. Petri dishes were placed in a refrigerator for one day to break dormancy. Dishes were then transferred to a growth chamber set at 22/20 °C day/night temperature and a 12 h photoperiod.

Na^+^ and K^+^ contents were determined following methods described previously^55^. Two-week-old seedlings were transferred to ½ MS containing 150 mM NaCl. Approximately 15–20 seedlings (0.05 g) were harvested after 0 d and 4 d of treatment. Tissues were kept at 105 °C for 20 min and dried at 80 °C to constant weight. Dry tissues were ground into a powder, and 1 ml 100 mM acetic acid was added. Samples were incubated at 90 °C for 6 h. Sodium and potassium contents were determined using an atomic absorption spectrometer with a continuous light source (ContrAA® 300, Analytik Jena AG). The analysis was repeated three times.

### Expression analysis of stress-responsive genes

Seeds of transgenic and WT *Arabidopsis* were handled as described above in “Determination of Na^+^ and K^+^ content.”

The expression of 13 stress-related genes listed in Supplementary Table S2 was determined in two-week-old seedlings. Three plants each from WT and every *Arabidopsis* transgenic lines (L-1, L-2, L-4) were harvested at around 10 O'clock. RNA was isolated from the leaves, and cDNA was synthesized through RT-PCR. Gene expression was determined through real-time PCR or qPCR. The analyses were repeated 2 times using each cDNA as a template. The *Actin* (NM_112764.3) gene was used as an internal control.

### Statistical analyses

Analyses were performed using SPSS version 14.0 statistical software (SPSS Inc., Chicago, IL). For all analyses, P-values <0.05 were considered statistically significant.

## Additional Information

**How to cite this article**: Xu, Z. *et al.* The nuclear protein GmbZIP110 has transcription activation activity plays important roles in the response to salinity stress in soybean. *Sci. Rep.* **6**, 20366; doi: 10.1038/srep20366 (2016).

## Supplementary Material

Supplementary Table1

Supplementary Table2
