# Supplementary material for: The nuclear protein GmbZIP110 has transcription activation activity and plays important roles in the response to salinity stress in soybean
Source: Sci Rep. 2016 Feb 3;6:20366. doi: 10.1038/srep20366 (PMC4738249; doi:10.1038/srep20366)
Supplement: Supplementary Table2 [file srep20366-s2.pdf]

The nuclear protein GmbZIP110 has transcription activation activity and plays important roles in the response to salinity stress in soybean

Zhaolong Xu[1, +], Zulfiqar Ali[2, +], Ling Xu[1], Xiaolan He[1], Yihong Huang[1], Jinxin Yi[1], Hongbo Shao[1*], Hongxiang Ma[1*] and Dayong Zhang[1*]

Table S2. List of primers of stress related genes and internal control

Gene	Locus	Forward primer (5′ - 3′)	Reverse primer (5′ - 3′)
CCA1	AT2G46830	TGTGGCTCAAACACTCCG	GCAATTCGACCCTCGTCA
LHY	AT1G01060	AAGTCTCCGAAGAGGGTC	CATGTTCCAACACCGATC
UGT71B6	AT3G21780	TTTGATGGAGCAAGACAG	GTTTCCGACCAAGCAATA
DREB2	AT5G05410	AACAGAAGGAGCAAGGGAT	ACATCGTCGCCATTTAGG
MYB2	AT2G47190	ACGCCCAATCATTACCCA	AACCTGACCCGTTCACCA
PAD3	AT3G26830	TATGCGATGGGTCGTGAT	TTTGGCTTCCTCCTGCTT
RCI3	AT1G05260	AGCCTCAACGATAACAAG	TGAAACAGACCTCTACGC
LTP3	AT5G59320	ATGTGGCACAGTGGCAGGTA	CTTGTTGGCGGTCTGGTG
LCL1	AT5G02840	TATTCCACCAGAAGATGA	CACTCCCAATGAAGTTAT
NHX1	AT5G27150	CCGTGCATTACTACTGGAGACAAT	GTACAAAGCCACGACCTCCAA
SOS1	AT2G01980	TCGTTTCAGCCAAATCAGAAAGT	TTTGCCTTGTGCTGCTTTCC
P5CS	AT3G55610	GAGGGGGTATGACTGCAAAA	AACAGGAACGCCACCATAAG
Actin	NM_112764.3	CCTCCGTCTTGACCTTGC	AGCGATACCTGAGAACATAGTG
